# Retraction Note: Association between placental site and successful induction of labor among postdate primiparous women

**DOI:** 10.1007/s00404-026-08399-y

**Published:** 2026-03-19

**Authors:** Omima T. Taha, Hanan M. Ghoneim, Tyseer Marzouk, Tamer Yehia M. Ali

**Affiliations:** 1 Department of Obstetrics and Gynecology, Faculty of Medicine, Suez Canal University, Ismailia, Egypt; 2 College of Applied Medical Science, University of Bisha, Bisha, Kingdom of Saudi Arabia; 3 Department of Woman’s Health and Midwifery Nursing, Faculty of Nursing, Mansoura University, Mansoura, Egypt

**Retraction Note: Archives of Gynecology and Obstetrics (2024) 311:661–667**

10.1007/s00404-024-07765-y

The Editor-in-Chief has retracted this article. Following publication, concerns were raised about the validity of the data reported. The authors provided a response to these concerns and their raw data, however post-publication statistical review determined that this response did not satisfactorily address the concerns raised and identified a number of further inconstancies in the data. The Editor-in-Chief has therefore lost confidence in the results and conclusions of this article.

Authors Omima T. Taha, Hanan M. Ghoneim, Tyseer Marzouk and Tamer Yehia M. Ali disagree with this retraction.

